# Supplementary material for: CSConv2d: A 2-D Structural Convolution Neural Network with a Channel and Spatial Attention Mechanism for Protein-Ligand Binding Affinity Prediction
Source: Biomolecules. 2021 Apr 27;11(5):643. doi: 10.3390/biom11050643 (PMC8145762; doi:10.3390/biom11050643)
Supplement: Supplementary file 1 [file biomolecules-11-00643-s001.zip › biomolecules-1183616-supplementary.pdf]

Supplementary Materials

A

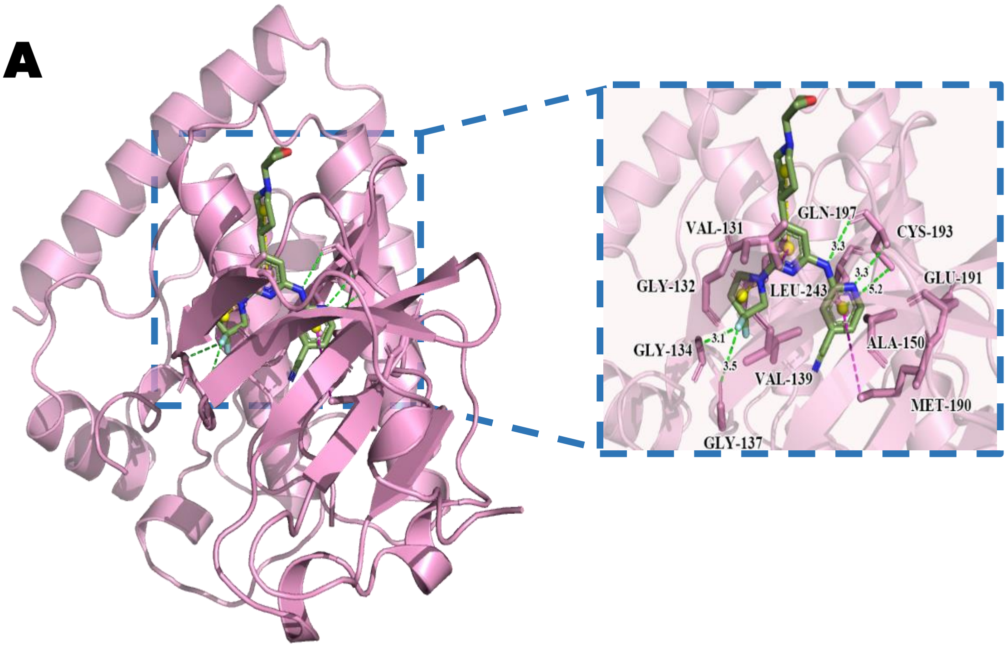

B

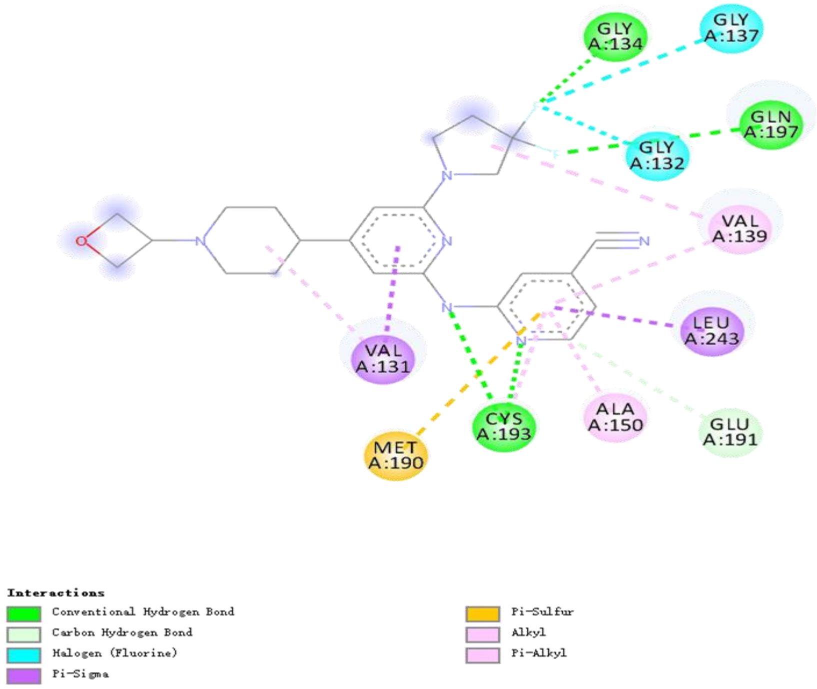

Figure S1. The interaction diagram between the DLK in complex with inhibitor 2-((6-(3,3-difluoropyrrolidin-1-yl)-4-(1-(oxetan-3-yl)piperidin-4-yl)pyridin-2-yl)amino)isonicotinonitrile (PDB ID: 5ceo) and 2-[[6-[3,3-bis(fluoranyl)pyrrolidin-1-yl]-4-[1-(oxetan-3-yl)piperidin-4-yl]pyridin-2-yl]amino]pyridine-4-carbonitrile (Chemical ID: 50D). (**A**) Molecular docking between the protein (PDB IDWx: 5ceo) and compound (Chemical ID: 50D). (**B**) Local two

dimensional display of the interaction diagram between the protein (PDB ID: 5ceo) and compound (Chemical ID: 50D).

Table S1. Results of molecular (kinase inhibitor) docking and predicted result by CSConv2d and DEEPScreen.

Target	Drug ID	Affinity (kcal/mol)	RMSD	Pred (CSConv2d)	Pred (DEEPScreen)	Label
mTOR	**DB00877**	**−18.0**	**0.3764**	**1**	**1**	**1**
	DB11651	−12.1	-	1	1	1
	DB13109	−11.0	-	1	0	1
	DB12986	−8.2	-	1	0	1
	DB12400	−7.6	-	1	0	1
VEGFR	**DB07334**	**−9.1**	**1.3344**	**1**	**0**	**1**
	DB06595	−10.3	-	1	1	1
	DB11977	−9.3	-	0	1	1
	DB14765	−8.0	-	1	0	1
	DB05608	−7.9	-	1	0	1
JAK	**DB08895**	**−8.9**	**1.2841**	**1**	**1**	**1**
	DB11763	−8.7	-	0	0	1
	DB12154	−8.4	-	1	1	1
	DB11817	−7.5	-	0	1	1
	DB14973	−6.8	-	1	0	1
